# Development of a reliable assay to measure glypican-1 in plasma and serum reveals circulating glypican-1 as a novel prostate cancer biomarker

**DOI:** 10.18632/oncotarget.25009

**Published:** 2018-04-27

**Authors:** Rachel A. Levin, Maria E. Lund, Quach Truong, Angela Wu, Neal D. Shore, Daniel R. Saltzstein, Raoul S. Concepcion, Thomas A. Paivanas, Arletta van Breda, Jennifer Beebe-Dimmer, Julie J. Ruterbusch, Sandra Wissmueller, Douglas H. Campbell, Bradley J. Walsh

**Affiliations:** ^1^ Minomic International Ltd, Sydney, New South Wales, Australia; ^2^ CUSP LLC Research Consortium, Annandale, VA, USA; ^3^ Barbara Ann Karmanos Cancer Institute and Wayne State University, School of Medicine, Department of Oncology, Detroit, MI, USA

**Keywords:** prostate cancer, biomarker, glypican-1, MIL-38, 3G5

## Abstract

Prostate cancer is responsible for hundreds of thousands of annual deaths worldwide. The current gold standard in early detection of prostate cancer, the prostate specific antigen test, boasts a high sensitivity but low specificity, resulting in many unnecessary prostate biopsies. Thus, emphasis has been placed on identifying new biomarkers to improve prostate cancer detection. Glypican-1 has recently been proposed as one such biomarker, however further exploration into its predictive power has been hindered by a lack of available, dependable glypican-1 immunoassays. Previously, we identified human glypican-1 as the antigenic target of the MIL-38 monoclonal antibody. Additionally, we have now generated another monoclonal antibody, 3G5, that also recognizes human glypican-1. Here we report the development of a reliable, custom Luminex^®^ assay that enables precise quantitation of circulating human glypican-1 in plasma and serum. Using this assay, we show for the first time that circulating glypican-1 levels can differentiate non-cancer (normal and benign prostatic hyperplasia) patients from prostate cancer patients, as well as benign prostatic hyperplasia patients alone from prostate cancer patients. Our findings strongly promote future investigation into the use of glypican-1 for early detection of prostate cancer.

## INTRODUCTION

Prostate cancer (CaP) is the second most common male cancer and fifth leading cause of male cancer deaths globally [1]. The highest incidence rates of CaP occur in Australia/New Zealand, North America, and Northern/Western Europe—largely due to the popularity of the prostate specific antigen (PSA) test that increases the number of prostate biopsies in these regions [1]. While the PSA test has a diagnostic sensitivity of ~80% for CaP, its specificity is only ~30% at the traditional PSA cutoff of 4 ng/mL [2, 3]. Since the majority of men with PSA >4 ng/mL do not have CaP, the PSA test is responsible for a high rate of unnecessary biopsies that can result in complications such as bleeding, infection, and transient urinary or erectile dysfunction [4]. Furthermore, the PSA test fails to differentiate patients with CaP from patients with benign prostatic hyperplasia (BPH)—a common non-cancerous enlargement of the prostate gland that raises PSA levels [5]. Thus, new biomarkers are urgently required to improve upon the PSA test [6, 7].

Recently, the heparan sulfate proteoglycan glypican-1 (GPC-1) has been highlighted as a potential protein biomarker for CaP [8]. GPC-1 is a co-receptor of tumorigenic heparin-binding growth factors, and its overexpression is associated with angiogenesis, metastasis, and poor prognosis [9, 10]. We have previously identified a cell-surface form (~55-60 kDa) and soluble, secreted forms (~40-52 kDa) of GPC-1 [8]. Modification of GPC-1 is controlled by enzymes such as heparanase (HSPE), amyloid precursor protein (APP), and amyloid precursor-like protein 2 (APLP2), as well as non-enzymatically by copper ions, nitric oxide, and ascorbate [11, 12].

We have previously determined GPC-1 to be the antigen recognized by the monoclonal antibody known as MIL-38 [8, 13]. We showed that MIL-38 could detect cell surface GPC-1 in CaP cell lysates and soluble GPC-1 in conditioned cell culture medium [8], suggesting that MIL-38 may also be used to measure soluble, circulating GPC-1 in human plasma and serum. Given the demonstrated potential of cell-surface GPC-1 as a CaP biomarker [13], we hypothesized that circulating GPC-1 levels may also differentiate patients with and without CaP.

Current commercial GPC-1 immunoassay kits utilize antibodies raised against human GPC-1 expressed by *E. coli*, which commonly folds and modifies proteins differently than eukaryotes [14]. These kits have failed to dependably detect recombinant, cell lysate, and soluble human GPC-1 in our hands, impeding further exploration into GPC-1 as a circulating CaP biomarker. To overcome this roadblock, here we present the development of a Luminex^®^ magnetic bead-based assay for measuring GPC-1 that employs MIL-38 as the capture antibody and a new monoclonal antibody developed by us, 3G5, as the detector antibody. Additionally, we describe the use of our novel assay to detect circulating GPC-1 in human plasma and serum and establish circulating GPC-1 as a new CaP biomarker.

## RESULTS AND DISCUSSION

### Monoclonal antibody characterization

**Figure 1:**
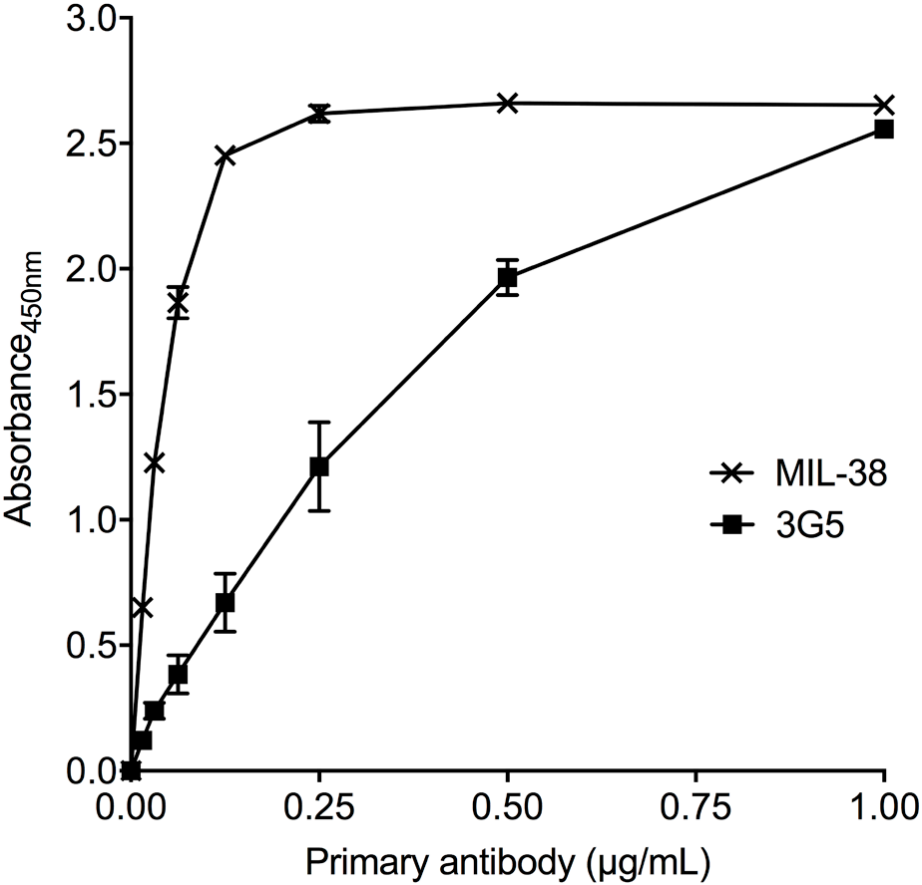
Recognition of recombinant human GPC-1 protein by MIL-38 and 3G5 Direct binding ELISAs of recombinant GPC-1 with serially diluted MIL-38 or 3G5 primary antibody (n = 3) and anti-mouse HRP secondary antibody. Primary antibody binding was measured as absorbance at 450 nm (mean ± SD) after 10 min of development.

**Figure 2:**
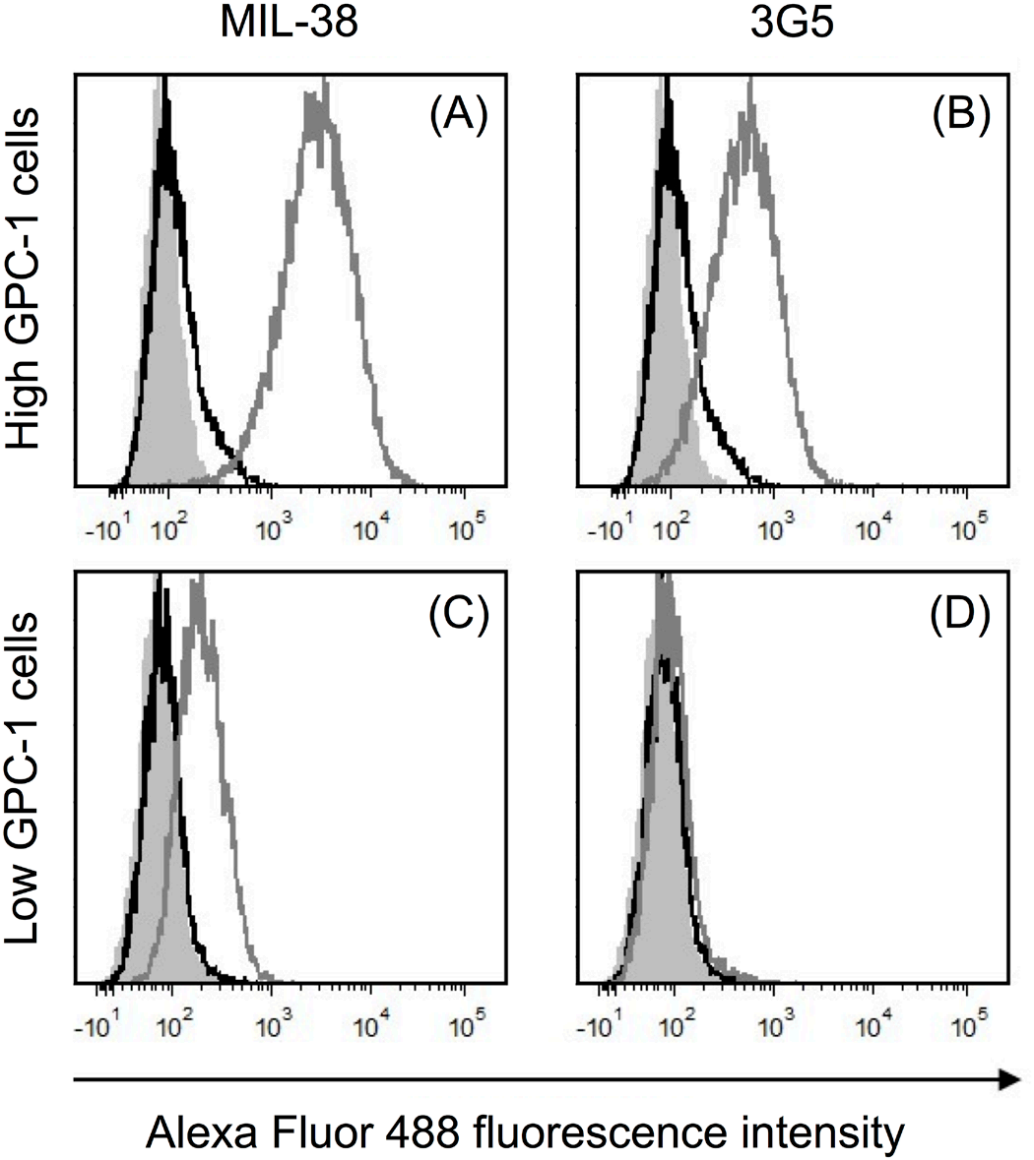
Recognition of cell-surface human GPC-1 protein by MIL-38 and 3G5 Flow cytometry histograms of fluorescence within the live cell gate for unstained cells (grey filled), no primary antibody control (black unfilled overlay) and primary antibody staining (grey unfilled overlay). Primary antibody binding was detected using anti-mouse IgG Alexa Fluor 488 secondary antibody. **(A)** High GPC-1 expressing cells stained with MIL-38 primary antibody (1 μg/mL). **(B)** High GPC-1 expressing cells stained with 3G5 primary antibody (50 μg/mL). **(C)** Low GPC-1 expressing cells stained with MIL-38 primary antibody (1 μg/mL). **(D)** Low GPC-1 expressing cells stained with 3G5 primary antibody (50 μg/mL).

MIL-38 and 3G5 binding to recombinant human GPC-1 was confirmed through direct binding ELISA (Figure 1). However, binding affinity of MIL-38 was higher than 3G5, indicating MIL-38 would be the preferred capture antibody for subsequent GPC-1 assay development. MIL-38 and 3G5 were also confirmed to bind cell-surface GPC-1 by flow cytometry. Both MIL-38 and 3G5 bound cells expressing high levels of GPC-1, while both antibodies had reduced binding to cells expressing low levels of GPC-1 (Figure 2). Again, greater binding to cell-surface GPC-1 was observed with MIL-38 compared to 3G5.

**Figure 3:**
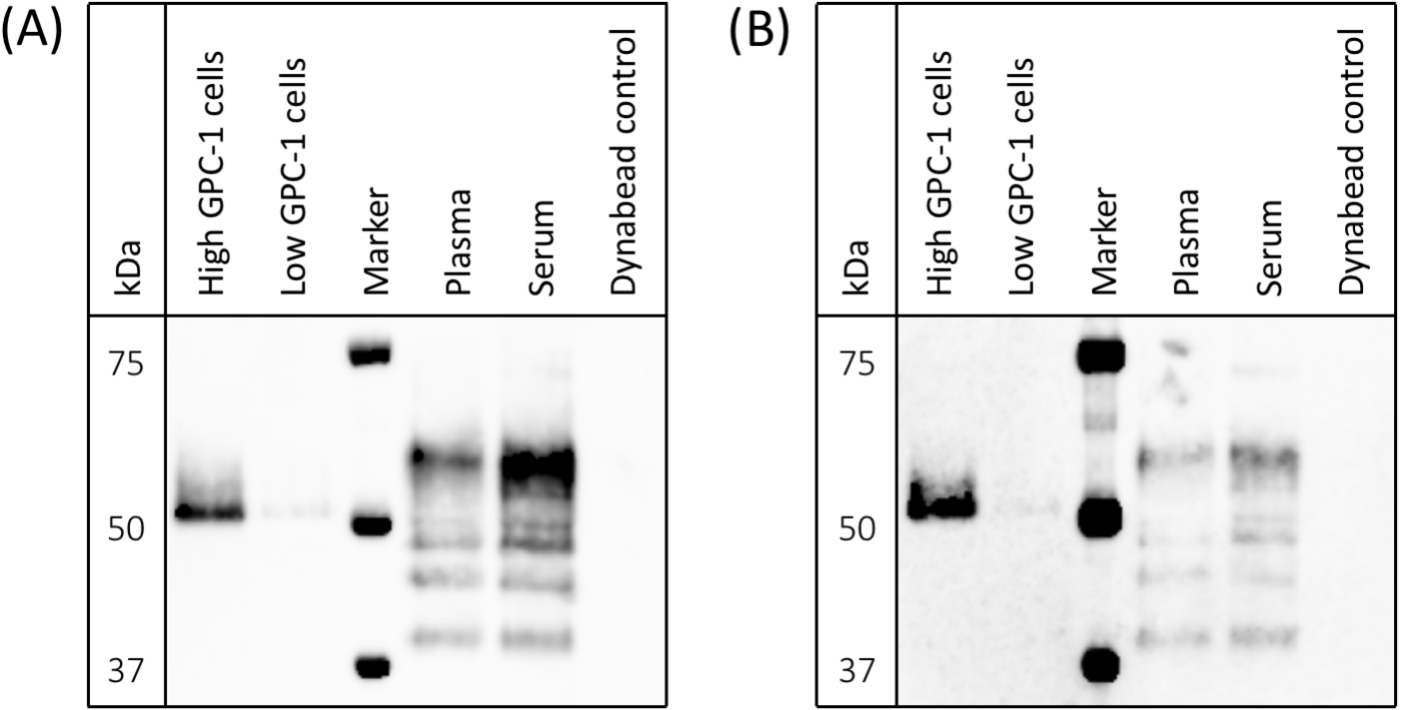
Detection of cell-surface and circulating GPC-1 isoforms by MIL-38 and 3G5 Western blots of immuno-precipitates from cell membrane protein extracts, plasma, serum, and Buffer SB (MIL-38-conjugated Dynabead® negative control). Precision Plus Protein Western C standard served as the protein marker. **(A)** GPC-1 isoforms detected using MIL-38 primary antibody; blot imaged with a 30 sec exposure. **(B)** GPC-1 isoforms detected using 3G5 primary antibody; blot imaged with a 2 min exposure.

MIL-38 and 3G5 each have different GPC-1 epitopes (patent submitted). To confirm 3G5 can specifically detect multiple GPC-1 isoforms as previously shown for MIL-38 [8], protein was immuno-precipitated from cell membrane extracts and from plasma and serum using MIL-38-conjugated Dynabeads^®^. Isolated protein was eluted and analyzed by western blot with MIL-38 primary antibody or 3G5 primary antibody (Figure 3). MIL-38 and 3G5 blots were equivalent: the cell-surface GPC-1 isoform was strongly detected only in membrane extracts of high GPC-1 expressing cells, and multiple circulating GPC-1 isoforms were detected in plasma and serum. No GPC-1 isoforms were detected in the MIL-38-conjugated Dynabead^®^ alone negative control. As expected, the circulating GPC-1 isoforms in plasma and serum differed from the cell-surface GPC-1 isoform due to post-translational modification and/or differential glycosylation [15, 16] and corresponded with the size range previously identified for those secreted in conditioned cell culture medium (~40-52 kDa) [8]. These results verified that MIL-38 and 3G5 can use different GPC-1 epitopes to detect GPC-1 isoforms in cell membrane extracts, plasma, and serum.

### GPC-1 Luminex^®^ assay validation and specifications

Preliminary experiments showed MIL-38 and 3G5 could act as a sandwich ELISA pair (data not shown). Accordingly, we developed a custom Luminex^®^ magnetic bead-based assay (an ELISA alternative) specifically for human GPC-1 using MIL-38 and 3G5. Magnetic bead-based immunoassays have previously been confirmed as a reliable and reproducible platform for measuring circulating proteins in CaP biofluid [17].

**Figure 4:**
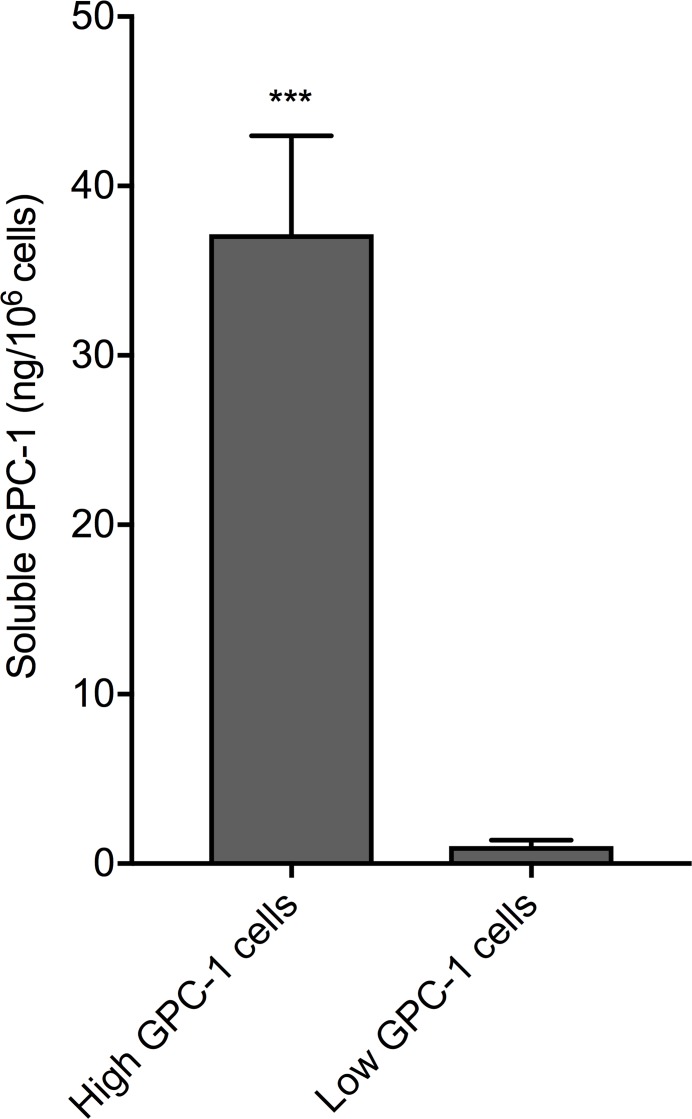
Recognition of soluble GPC-1 protein in cell culture medium using the MIL-38/3G5 Luminex^®^ assay GPC-1 in RPMI-1640 medium without FBS from low GPC-1 expressing cells and high GPC-1 expressing cells (n = 3) was measured using the Luminex^®^ assay with MIL-38 capture antibody and 3G5 detection antibody. GPC-1 concentrations (ng/mL) were normalized by cell density (10^6^ cells/mL) to calculate soluble GPC-1 per 10^6^ cells (mean ± SD). Statistical significance (^***^p = 0.0004) was determined by unpaired t-test.

A 6-point serial (1:3) dilution of recombinant human GPC-1 standard was measured with a prototype of the GPC-1 Luminex^®^ assay by the assay manufacturers, R&D Systems Custom Services (a division of Bio-Techne, MN, USA), and by us at the Australian Proteome Analysis Facility (Sydney, AU). Mean fluorescence intensity (MFI, background-subtracted) was highly correlated (R^2^ = 0.99, slope = 1.01), verifying assay reproducibility by different operators in different laboratories. The finalized GPC-1 Luminex^®^ assay included a 7-point recombinant human GPC-1 standard curve and had low background fluorescence (≤15 MFI). The assay had a background-subtracted MFI signal range of 4 units to 5,800 units for GPC-1 concentrations of 0.15 - 111 ng/mL. Reliable quantitation of soluble GPC-1 by the Luminex^®^ assay was shown by measuring conditioned Roswell Park Memorial Institute (RPMI-1640) medium (Sigma) from cultures of cells with either high or low GPC-1 protein expression levels (Figure 4); soluble GPC-1 was significantly elevated in medium from cells with high GPC-1 levels relative to medium from cells with low GPC-1 levels (p = 0.0004, unpaired t-test). Furthermore, mean soluble GPC-1 in commercially available human serum (Cat no. CR200-M-1L, TCS Biosciences) measured in duplicate across different Luminex^®^ assay runs in this study produced concentration values with <1% CV (mean ± SD; 21.56 ± 0.37 ng/mL versus 21.44 ± 1.44 ng/mL).

### Patient demographic

Normal, BPH, and CaP patients in the United States were recruited for this study (Table 1) and provided matched samples of plasma and serum. Patients were predominantly Caucasian. Age and body mass index (BMI) were not statistically different between between non-CaP (normal and BPH) patients and CaP patients (p = 0.18, unpaired t-test) or between BPH patients and CaP patients (p = 0.92, unpaired t-test). PSA could not be used to compare patient cohorts without bias since a low PSA threshold (<2ng/mL for patients ≤60 years of age, <3ng/mL for patients >60 years of age) was set for the normal cohort, whereas no PSA restriction or testing was required for the BPH or CaP cohort.

**Table 1:** Clinical data

	Normal patients (n = 15)	BPH patients (n = 15)	CaP patients (n = 15)
**Age in years (range, median)**	51-78, 62	54-80, 69	53-81, 68
**BMI (range, median)**	23-45, 28	23-36, 29	21-43, 28
**PSA ng/mL (range, median)**	0.27-2.83, 1.17	0.17-8.71, 3.95 ^*^	3.6-27.7, 8.26
**Gleason grading (score, n)**	-	-	4+3, 3 4+4, 7 4+5, 3 5+4, 2

^*^ PSA data not available for 4 patients

### Circulating human GPC-1 as a CaP biomarker

**Figure 5:**
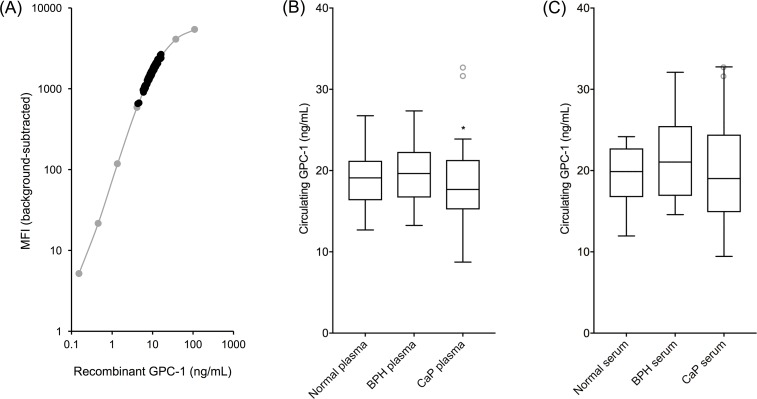
Circulating human GPC-1 as a CaP biomarker MIL-38/3G5 Luminex^®^ assay measurements for circulating GPC-1 protein. **(A)** Recombinant GPC-1 standard curve (grey) overlayed with circulating GPC-1 measurements from plasma and serum samples (black). **(B)** Tukey plot of circulating GPC-1 in plasma samples from normal, BPH, and CaP patients (n = 15). **(C)** Tukey plot of circulating GPC-1 in matched serum samples from the same normal, BPH, and CaP patients (n = 15). Tukey plots determined two outliers in the CaP plasma samples and no outliers in the serum samples. Outlier CaP plasma samples, along with the matched serum samples from the same patients, are shown as grey, unfilled circles. Statistical significance for non-CaP plasma vs CaP plasma (^*^p = 0.0483) and for BPH plasma vs CaP plasma (^*^p = 0.0473) was determined by unpaired t-tests with outliers removed.

Circulating GPC-1 ranged from 8.74 - 32.67 ng/mL in plasma and 9.44 - 32.76 ng/mL in serum (Figure 5A). Plasma and serum GPC-1 concentrations were generally correlated (R^2^ = 0.78, slope = 0.97) and had comparable trends for medians across cohorts, though the distribution of GPC-1 concentrations within each cohort was tighter in plasma (Figure 5B) than in serum (Figure 5C). Two outliers were identified in the plasma samples using box-and-whisker Tukey plots [18] and were removed for statistical analyses. No outliers were identified in the serum samples due to the broader distribution of GPC-1 concentrations within each cohort.

Circulating GPC-1 data followed a normal distribution. GPC-1 concentration means ± SD for normal, BPH, and CaP plasma samples were 19.00 ± 3.85 ng/mL, 19.98 ± 3.74 ng/mL, and 16.86 ± 4.19 ng/mL, respectively. GPC-1 concentration medians for normal, BPH, and CaP plasma samples were 19.10 ng/mL, 19.64 ng/mL, and 17.32 ng/mL, respectively. GPC-1 concentration means ± SD for normal, BPH, and CaP serum samples were 19.44 ± 3.81 ng/mL, 21.21 ± 5.13 ng/mL, and 20.10 ± 6.74 ng/mL, respectively. GPC-1 concentration medians for normal, BPH, and CaP serum samples were 19.87 ng/mL, 21.05 ng/mL, and 19.02 ng/mL, respectively.

Plasma GPC-1 concentrations were significantly different for non-CaP patients versus CaP patients (p = 0.0483, unpaired t-test), as well as for BPH patients alone versus CaP patients (p = 0.0473, unpaired t-test). Serum GPC-1 concentrations were not statistically different between patient cohorts due to broader GPC-1 concentration distributions. GPC-1 in both plasma and serum was not collinear with PSA (R^2^ < 0.02, slope < 0.2), enhancing potential for their joint utility to improve early detection of CaP over the use of PSA alone [6, 19].

Although cell-surface GPC-1 overexpression is associated with CaP [13], circulating GPC-1 was reduced in CaP patients. However, divergent regulation of cell-surface GPC-1 and circulating GPC-1 may explain this discrepancy since abnormal protein secretion is characteristic of cancer cells [20]. Moreover, localization and shedding of heparan sulfate proteoglycans such as GPC-1 is controlled by multiple enzymes including HSPE [12, 15]. Interestingly, both *GPC-1* and *HSPE* genes are down-regulated in the transcriptomes of primary prostate adenocarcinoma tumors (NCI Genomic Data Commons, https://gdc.cancer.gov/) (Supplementary Figure 1). Reduced *HPSE* expression preserves heparan sulfate side chains present on the core proteoglycan, which suppress extracellular protein shedding [21]. Additionally, *APP* and *APLP2* genes that modulate GPC-1 protein degradation are transcriptionally dysregulated (down-regulated and up-regulated, respectively) in primary prostate adenocarcinoma tumors (Supplementary Figure 1). While mRNA and protein levels may only be loosely correlated [22], differential transcription of *GPC-1*, *HSPE*, *APP*, and *APLP2* highlights the complex nature of GPC-1 protein expression, modification, and localization that may contribute to increased cell-surface GPC-1 and reduced circulating GPC-1 in CaP patients.

In this study, we have developed a novel Luminex^®^ assay for measuring human GPC-1 in conditioned cell culture medium, plasma, and serum. Using this assay, we reveal circulating GPC-1 as a new CaP biomarker. For the first time, we show GPC-1 levels in plasma significantly differentiate non-CaP from CaP patients and BPH patients specifically from CaP patients, which is a major shortcoming of the PSA test [5]. Our results have motivated a larger follow-up study of 300 patients that is currently being analyzed to further evaluate GPC-1 as an important biomarker for early detection of CaP.

## MATERIALS AND METHODS

### Monoclonal antibodies and recombinant human GPC-1

MIL-38 monoclonal antibody was produced from hybridoma cell stocks as previously described [8]. The mouse 3G5 monoclonal antibody was generated by immunization against a specific GPC-1 peptide sequence distinct from the MIL-38 epitope (patent submitted) and purified from stable hybridoma cell stocks by Genscript (NJ, USA). Recombinant human GPC-1 protein was produced by murine NS0 cells (Cat no. 4519-GP, R&D Systems).

### Cell lines and culture conditions

A high GPC-1 expressing cell line (DU-145, CaP) was purchased from ATCC, and a low GPC-1 expressing cell line (C3, bladder cancer) [13] was provided by Professor Pamela Russell (Australian Prostate Cancer Research Centre, Institute of Health and Biomedical Innovation, Queensland University of Technology, Australia). Cells were grown in triplicate T25 flasks (Greiner Bio-One) in R10 medium (RPMI-1640 medium supplemented with 10% v/v fetal bovine serum, FBS). Once cultures reached 60-70% confluency, cells were collected for analysis by flow cytometry by incubation in PBS with 2 mM EDTA (15 min, 37°C/5% CO_2_).

In parallel, both cell lines were grown to 80% confluency in additional triplicate T25 flasks. R10 medium was then replaced with RPMI-1640 medium without FBS. After 18 hr, conditioned medium was collected and centrifuged at 200 × *g* for 5 min to remove any suspended cells. The supernatant was then stored at -80°C. Adherent cells were detached by incubation in PBS with 2 mM EDTA (15 min, 37°C/5% CO_2_), pooled with any pelleted suspended cells, and counted using a haemocytometer.

### Direct binding ELISA

Wells of a clear flat-bottom Nunc MaxiSorp 96-well plate (Cat no. 439454, Thermo Fisher Scientific) were coated with 300 μL of 10 mM sodium carbonate buffer (pH 9) and left at room temperature for 15 min. Buffer was then replaced with 300 μL of fresh sodium carbonate buffer containing 0.5 μg/mL of recombinant human GPC-1 protein. After an overnight incubation at room temperature, sodium carbonate buffer with GPC-1 was removed. Wells were blocked with 300 μL of casein in PBS (Cat no. 37528, Thermo Fisher Scientific) at room temperature for 1 hr. Next, blocking solution was replaced with primary antibody (either MIL-38 or 3G5) that was serially (1:2) diluted from 1 μg/mL down to 0.0078 μg/mL in PBS with 0.1% TWEEN 20 (PBS-T) and 10% casein (PBS-T/C). After a 1 hr incubation at room temperature, primary antibody was removed and wells were washed four times with 300 μl of PBS-T. Sheep anti-mouse IgG-HRP secondary antibody (Cat No. NXA931, GE Healthcare) was then diluted 1:2,000 in PBS-T/C and added to wells. Following another 1 hr incubation at room temperature, wells were washed four times with 300 μL of PBS-T, and 100 μL of 3, 3′, 5, 5′-Tetramethylbenzidine (TMB) Liquid Substrate (Cat no. T4444, Sigma) was added to each well. The reaction was left to develop for 10 min before adding 300 μL of Stop Reagent for TMB Substrate (Cat no. S5814, Sigma). The plate was read at λ_450nm_ using an xMark Microplate Absorbance Spectrophotometer (BioRad).

### Flow cytometry

MIL-38 antibody was diluted to a working concentration of 1 μg/mL in FACS Staining Wash (FSW) made up of PBS pH 7.4 supplemented with 2% FBS. 3G5 antibody was diluted to a working concentration of 50 μg/mL in FSW. The high and low GPC-1 expressing cells (4 × 10^5^) were stained with 50 μL of diluted MIL-38 or 3G5 on ice for 45 min. Cells were washed twice with 800 μL of FSW by centrifugation at 300 × *g*. The washed cells were then stained with 50 μL of goat anti-mouse IgG Alexa Fluor 488 (Cat no. A11029, Thermo Fisher Scientific; 20 μg/mL diluted in FSW) for 45 min on ice protected from light. After staining, cells were washed three times with 800 μL of FSW. Topro-3-iodide (Thermo Fisher Scientific; 0.1 μM) was added to allow discrimination of viable cells and data was acquired using a BD Fortessa X20 Flow Cytometer (BD Biosciences). Analysis was performed using the FCS Express 5 Flow Research Edition software package (De Novo Software). Analyses were restricted to live single cells.

### Membrane protein extraction, immuno-precipitation, and western blotting

Both cell lines were harvested from tissue culture flasks by incubation in PBS with 2 mM EDTA (15 min, 37°C/5% CO_2_), pelleted at 1000 × *g* for 10 min and stored in PBS at -80°C. To extract membrane proteins, high GPC-1 expressing cells (4 × 10^7^) and low GPC-1 expressing cells (6 × 10^7^) were thawed at 37°C then pelleted at 1000 × *g* for 10 min. Protease inhibitor cocktail (Cat no. P8340, Sigma-Aldrich; 40 μL) was added to the pellet. The pellet was immediately resuspended in 8 mL of Buffer I (20 mM MES pH 6.5, 0.5 mM EDTA, 0.005% Triton X100; pH 6.5) and incubated on a rotating mixer for 10 min. Samples were pelleted at 16,000 × *g* for 15 min. Protease inhibitor cocktail (20 μL) was added to the pellet, which was then resuspended in 4 mL of Buffer II (20 mM HEPES pH 7.5, 0.5 mM EDTA, 0.5% Triton X100; pH 7.5) and incubated on a rotating mixer for 10 min. Again, samples were pelleted at 16,000 × *g* for 15 min. The supernatant was collected and stored at -20°C.

MIL-38 was coupled to magnetic Dynabeads^®^ and washed using the Dynabeads^®^ Antibody Coupling Kit (Cat no. 14311D, Thermo Fisher Scientific; 5 μg MIL-38 per 1 mg Dynabeads^®^). MIL-38-coupled Dynabeads^®^ were then resuspended in Buffer SB (Thermo Fisher Scientific, 50 μL per mg beads). Cell-bound protein extract (2 mL) was mixed with 1.5 mg of MIL-38-coupled Dynabeads^®^ on a rotating mixer for 3 hrs at room temperature. Pooled plasma (barcode 837361, Australian Red Cross Blood Service) and pooled serum (Cat no. CR200-M-1L, TCS Biosciences) were centrifuged at 20,000 × *g* for 5 min to remove any cellular debris. The supernatant (2 mL) from each was mixed with 3 mg of MIL-38-coupled Dynabeads^®^ on rotating mixer for 1 hr at room temperature then exchanged with a new 2 mL of plasma and serum. This was repeated until 10 mL total of plasma and serum was used. MIL-38-coupled Dynabeads^®^ (1 mg) in Buffer SB served as a negative control. MIL-38-coupled Dynabeads^®^ were then washed four times with 1 mL PBS-T. Bound protein was eluted with 65 μL of IgG Elution Buffer (Cat no. 21009, Thermo Fisher Scientific).

Eluate (21 μL) was mixed with 4× XT sample buffer (Cat no. 161-0791, BioRad; 7 μL) and boiled for 5 min at 95°C. The samples (28 μL), along with Precision Plus Protein Western C standards (Cat no. 161-0376, BioRad; 2 μL), were then loaded into 4-12% Bis-Tris Plus gels (Cat no. NW04127B0X, Thermo Fisher Scientific) and run at 200 V in MOPS SDS running buffer (Cat no. B0001-02, Thermo Fisher Scientific). Protein was transferred from the gel to a nitrocellulose membrane (Cat no. 1704159, BioRad) at 25 V for 10 min using a Trans-Blot Turbo (BioRad). The membrane was then blocked with 5% skim milk/PBS-T overnight at 4°C on a rocking platform.

To confirm no non-specific binding of secondary protein, the membrane was incubated with Pierce™ High Sensitivity Streptavidin-HRP (Cat no. 2113, Thermo Fisher Scientific; 1:20,000 dilution) and StrepTactin-HRP Conjugate (Cat no. 161-0380, BioRad; 1:10,000 dilution) in 5% skim milk/PBS-T for 30 min at room temperature on rocking platform. The membrane was then washed three times with PBS-T. The membrane covered with Clarity Western ECL substrate (BioRad) for 1 min then imaged with a ImageQuant LAS4000 mini (GE Life Sciences).

ECL substrate was removed from the membrane with three washes of PBS-T, and the membrane was incubated with biotinylated MIL-38 primary antibody (1 μg/mL) or biotinylated 3G5 primary antibody (10 μg/mL) in 5% skim milk/PBS-T for 1 hr. The membrane was washed three times with PBS-T then incubated with Streptavidin-HRP and StrepTactin-HRP secondary proteins in 5% skim milk/PBS-T, as described earlier. Finally, the membrane was washed six times with PBS-T, covered with ECL substrate for 1 min, then imaged with a ImageQuant LAS4000 mini (GE Life Sciences).

### GPC- 1 Luminex^®^ assay development

The GPC-1 Luminex*^®^* assay was manufactured by R&D Systems Custom Services. MagPlex^®^ Microspheres (Luminex Corporation), bead region 18, were covalently coupled with MIL-38 capture antibody using xMAP^®^ Technology (Luminex Corporation) according to the manufacturer's protocol (xMAP^®^ Cookbook, 3^rd^ Edition). 3G5 detection antibody was biotinylated by R&D Systems Custom Services using standard protocols. Recombinant human GPC-1 (Cat no. 4519-GP, R&D Systems) was used as the protein standard for the Luminex*^®^* assay. Duplicate aliquots of pooled male human serum (Cat no. CR200-M-1L, TCS Biosciences) were measured on each run of the GPC-1 Luminex^®^ assay to serve as an inter-run biofluid control.

### Patient population and sample processing

Forty-five patients comprising three cohorts were recruited in the United States. The normal patient cohort consisted of 15 males, ≥50 years of age that had a normal digital rectal examination and a low, age-adjusted serum PSA (<2ng/mL for patients ≤60 years, <3ng/mL for patients >60 years of age). PSA eligibility for patient recruitment was based on previous PSA test results from 1 week to 1 year prior to the study. The BPH patient cohort consisted of 15 males, ≥50 years of age with pathological BPH that was confirmed by transurethral resection of the prostate 4 weeks to 1 year prior to this study. The CaP patient cohort consisted of 15 males ≥50 years of age that had biopsy-confirmed CaP with a Gleason score of at least 7 (≥Gleason 3+4).

Blood specimens for plasma were collected in 10 mL lavender top Plasma EDTA Vacutainers (Becton Dickinson) then placed on ice. Blood specimens for serum were collected in 10 mL red top Serum Plus Vacutainers (Becton Dickinson) then left to clot at room temperature. Both serum and plasma were processed between 30 min to 2 hr after collection through centrifugation at 1,300 × *g* for 15 min. Plasma and serum samples were stored at -20°C until shipment to Minomic International Ltd (NSW, AU) where they were stored long-term at -80°C. This study was approved by the Western Institutional Review Board (IRB), in accordance with 21CFR56. Informed consent was obtained from each patient.

### Measurement of soluble GPC-1

Conditioned RPMI-1640 medium, human plasma samples, and human serum samples were thawed at 4°C then centrifuged at 20,000 × *g* for 5 min at room temperature to pellet any cellular debris. Sample supernatants (75 μL) were diluted 1:2 in Calibrator Diluent RD6-40 (part #895817, R&D Systems). Diluted sample (150 μL) was added to each well of a 96-well plate (Eppendorf) according to a randomized plate plan. Sample plates were sealed and frozen at -80°C.

When ready to run the custom GPC-1 Luminex^®^ assay, recombinant GPC-1 standard was reconstituted in 0.5 mL of Calibrator Diluent RD6-40 (110.9 ng/mL GPC-1 working concentration) and mixed by vortexing. Standard was then left to sit at room temperature for a minimum of 15 min with occasional, gentle agitation. Meanwhile, the sample plate was thawed at room temperature on an orbital shaker at 800 rpm then sonicated for 10 min in a water bath. The reconstituted GPC-1 standard was serial (1:3) diluted to generate a 7-point standard curve. Each dilution of GPC-1 standard (50 μL), along with a blank (50 μL of Calibrator Diluent RD6-40) was added in duplicate to the first two columns of a black-walled 96-well plate (Greiner Bio-One). Thawed, diluted samples were mixed by pipetting and added to the remaining wells of the black-walled 96-well plate. MagPlex^®^ Microspheres (500 μL) coupled with MIL-38 were diluted in 5 mL of Microparticle Diluent (part #895529, R&D Systems), resuspended by vortexing, and aliquoted (50 μL) into all wells of the 96-well plate. The plate was sealed and incubated at room temperature on an orbital shaker at 800 rpm for 2 hr.

Following incubation, the plate was placed on a magnetic 96-well plate washer (BioRad) and washed three times with 100 μL Wash Buffer (part #895003, R&D Systems). Biotinylated 3G5 (50 μL) was added to each well, then the plate was sealed and incubated at room temperature on an orbital shaker at 800 rpm for 1 hr. The plate was washed as previously described, and 50 μL of Streptavidin-PE (part #892525, R&D Systems) was added to each well. The plate was sealed and incubated at room temperature on an orbital shaker at 800 rpm for 30 min, then the wash step was repeated. Samples were resuspended in 100 μL of Wash Buffer. The plate was sealed and incubated at room temperature on an orbital shaker at 800 rpm for 2 min. Finally, the plate was read using the Bio-Plex^®^ 200 system (BioRad) with a 50 event/bead count threshold and doublet discriminator gates set to 8000 and 17,500.

## SUPPLEMENTARY MATERIALS FIGURES AND TABLES


