# Supplementary material for: Development of a reliable assay to measure glypican-1 in plasma and serum reveals circulating glypican-1 as a novel prostate cancer biomarker
Source: Oncotarget. 2018 Apr 27;9(32):22359–67. doi: 10.18632/oncotarget.25009 (PMC5976470; doi:10.18632/oncotarget.25009)
Supplement: Supplementary file 1 [file oncotarget-09-22359-s001.pdf]

# Development of a reliable assay to measure glypican-1 in plasma and serum reveals circulating glypican-1 as a novel prostate cancer biomarker

## SUPPLEMENTARY MATERIALS

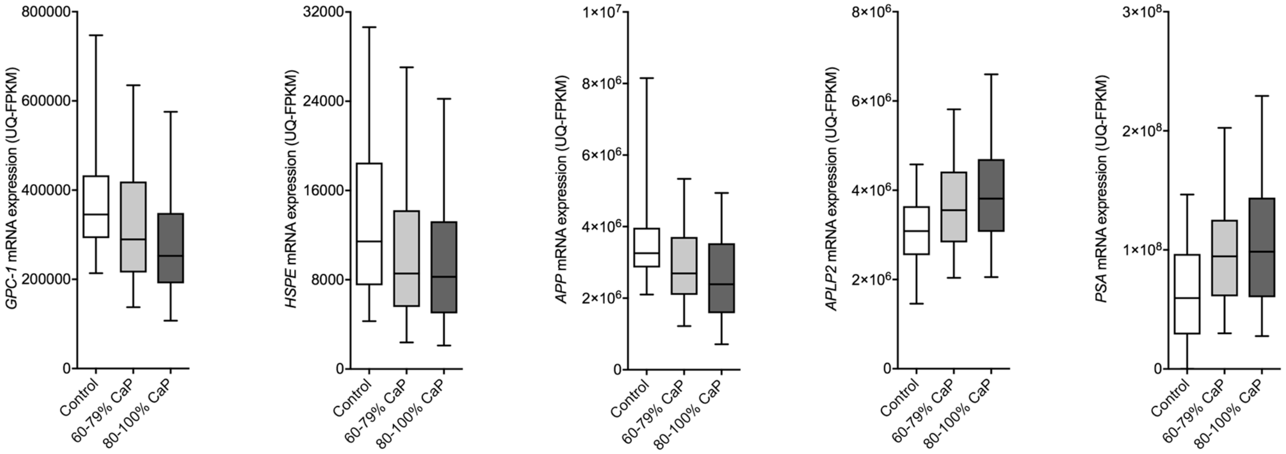


**Supplementary Figure 1: Transcriptional dysregulation in CaP.** Box-and-whisker plots ($5^{th}$-$95^{th}$ percentile) of gene expression in non-cancerous prostate tissue (control, n = 52), primary prostate tumor tissue with 60-79% positively stained cancer nuclei (60-79% CaP, n = 286), and primary prostate tumor tissue with 80-100% positively stained cancer nuclei (80-100% CaP, n = 226). Normalized RNA-Seq expression data (UQ-FPKM) was downloaded from the NCI Genomic Data Commons data portal (https://portal.gdc.cancer.gov/, Data Release 4.0).
